# Transcription factor TabHLH49 positively regulates dehydrin *WZY2* gene expression and enhances drought stress tolerance in wheat

**DOI:** 10.1186/s12870-020-02474-5

**Published:** 2020-06-05

**Authors:** Hao Liu, Ying Yang, Dandan Liu, Xiaoyu Wang, Linsheng Zhang

**Affiliations:** 1 College of Life Science/State Key Laboratory of Crop Stress Biology for Arid Areas, Northwest A&F University, Yangling, 712100 China; 2 College of Nursing, Weinan Vocational&Technical College, Weinan, 714000 China; 3 School of Agriculture, Yunnan University, Kunming, 650000 China; 4 Institute of Evolution & Marine Biodiversity, Ocean University of China, Qingdao, 266000 China

**Keywords:** Wheat, Dehydrin, Drought stress, bHLH transcription factor, Regulation mechanism

## Abstract

**Background:**

As functional proteins, dehydrins are found in many maturing seeds and vegetable tissues under adverse environmental conditions. However, the regulation of dehydrin expression remains unclear.

**Results:**

In this study, a novel drought stress-related bHLH transcription factor, TabHLH49, was isolated from a wheat cDNA library treated with the drought and cold stress by using yeast one-hybrid system. TabHLH49 protein possesses a typical conserved bHLH domain and is a Myc-type bHLH transcription factor. TabHLH49 was detected in the nucleus of tobacco epidermal cells, and the amino acid sequences at the C-terminus (amino acids 323–362) is necessary for its transactivation activity. Real-time PCR analyses revealed the tissue-specific expression and drought stress-responsive expression of *TabHLH49* in wheat. In addition, the verification in Y1H and electrophoretic mobility shift assays illustrated that TabHLH49 protein can bind and interact with the promoter of the wheat *WZY2* dehydrin. Furthermore, the dual-luciferase assays showed that TabHLH49 can positively regulate the expression of WZY2 dehydrin. The transient expression and BSMV-mediated gene silencing of TabHLH49 also showed that TabHLH49 positively regulates the expression of WZY2 dehydrin and improves drought stress resistance in wheat.

**Conclusions:**

These results provide direct evidences that TabHLH49 positively regulates expression level of dehydrin *WZY2* gene and improves drought tolerance of wheat.

## Background

As an important food crop, wheat has been widely cultivated in most parts of northern China. However, the plants typically suffer from the different types of abiotic stresses including drought, cold damage, or salt injury during their growth and development periods, which can directly affect the overall wheat production.

As sessile organisms, plants have evolved the ability to synthesize a series of stress-responsive proteins to avoid or defend against adverse conditions. Late embryogenesis abundant (LEA) proteins act as functional proteins in helping to protect cells from direct dehydration. Based on their conserved sequence motifs, the LEA proteins have been grouped into seven distinctive groups [1]. Group 2 LEA proteins, also known as dehydrins, are characterized by their highly hydrophilicity. They are considered hydrophiles and contain three typical conserved motifs: K-(EKKGIMDKIKEKLPG), Y-([V/T]D [E/Q]YGNP) and S-(serine-track) segments. Based on the presence of these conserved sequences (K-, Y-, and S-segments), dehydrins are classified into the different subclasses: YnKn, YnSKn, KnS, SKn and Kn [2]. The wheat dehydrin *WZY2* investigated in the current study is a YSK2-type dehydrin belonging to the YnSKn subtype [3].

The basic helix-loop-helix (bHLH) proteins are the second largest superfamily of plant transcription factors and can play important roles in hormone signaling, photomorphogenesis, and secondary metabolism. This family is well known due to its conserved basic-helix-loop-helix structural domain consisting of two functional regions, the basic amino acid region and the helix-loop-helix region (HLH) located at the N-terminus and C-terminus of the bHLH domain, respectively. The basic amino acid region contains basic residues that can recognize and specifically bind the DNA motif in target genes [4]. The helix-loop-helix region consists of two amphipathic alpha helices linked by a loop with variable length and amino-acid composition, which can promote the formation of homodimers or heterodimers to control gene transcription [5].

According to previous studies on the bHLH proteins, these plant transcription factors are widely involved in the regulation of plant physiological and metabolic pathways. For example, bHLH factors play an important biological role in the regulatory network controlling anther development in *Arabidopsis thaliana* [6, 7]. In addition, bHLHs are also involved in plant responses to drought, salinity, and chilling abiotic stress. AtbHLH112, a nuclear-localized protein induced by salt, drought and abscisic acid (ABA), encodes a bHLH protein. AtbHLH112 serves as a transcriptional activator that regulates the expression of genes via binding to their GCG- or E-boxes to mediate physiological responses, including proline biosynthesis and ROS scavenging pathways, to enhance stress tolerance [8]. Moreover, a bHLH gene ThbHLH1 from *Tamarix hispida* can improve abiotic stress tolerance by enhancing osmotic potential and decreasing reactive oxygen accumulation [9].

Many previous studies, including our studies, have shown that wheat synthesizes dehydrins when under abiotic stress. However, the upstream regulatory mechanism and function of dehydrins thus far remains unclear. In this study, we have further explored the stress response mechanism of the *WZY2* (UniProt ID: B0LXL4) dehydrin based on our cloned promoter of *WZY2* from wheat “*ZhengYin 1#*” in water stress. *TabHLH49* (UniProt ID: A0A3B6RCI9) was isolated and identified in the wheat cDNA library treated with drought and cold stress by using the yeast one-hybrid system. TabHLH49 protein encodes a basic helix-loop-helix (bHLH) transcription factor that functions as a positive regulator of *WZY2* dehydrin gene expression. This is the first detailed report of a bHLH involved in the mechanism of dehydrin regulation under abiotic stress and clarifies its role in the *WZY2* dehydrin signaling pathway.

## Results

### Characterization of TabHLH49 and bioinformatics analysis

**Fig. 1:**
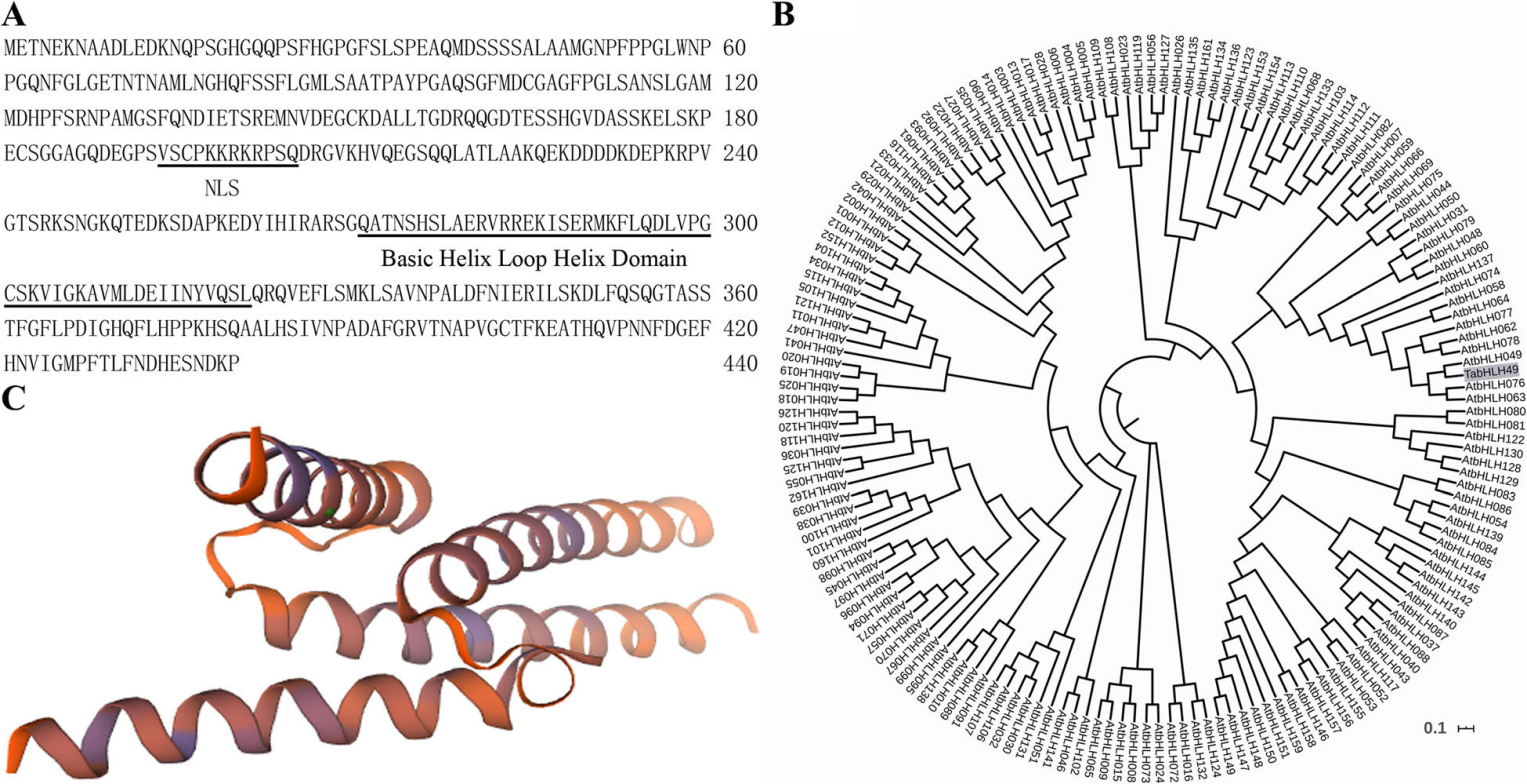
Sequence (**a**), phylogenetic tree (**b**) and three-dimensional structure (**c**) of TabHLH49 amino acids. The phylogenetic tree was constructed by the neighbor-joining method using MEGA7 software. Bootstrapping with 1000 replicates was used to assess the statistical reliability of nodes in the tree. The three-dimensional structure was created using the transcription factor MYC2 conserved domain structure as a template (ID: 5gnj.3.B, 99.6% sequence identity)

In our previous study, we isolated a cDNA segment of bHLH49-like protein interacting with dehydrin *WZY2* promoter performed by yeast one-hybrid (Y1H) assay [10], the complete coding sequence was obtained via a BLAST search against the wheat genomic database, and this gene was named TabHLH49. TabHLH49 encoded 440 amino acids with a predicted protein molecular weight of 47.54 kDa and an isoelectric point of 5.61 (Fig. 1a). This gene contained a conserved bHLH domain and belonged to the bHLH transcription factor family. The three-dimensional structure of TabHLH49 was constructed by SWISS-MODLE software (Fig. 1c). The phylogenetic tree was constructed using TabHLH49 and 162 bHLH gene sequences from *Arabidopsis thaliana* (Fig. 1b). The results indicated that TabHLH49 was highly homologous to AtbHLH049 and was a Myc-type bHLH transcription factor.

### Nuclear localization and transactivation activity of TabHLH49

**Fig. 2:**
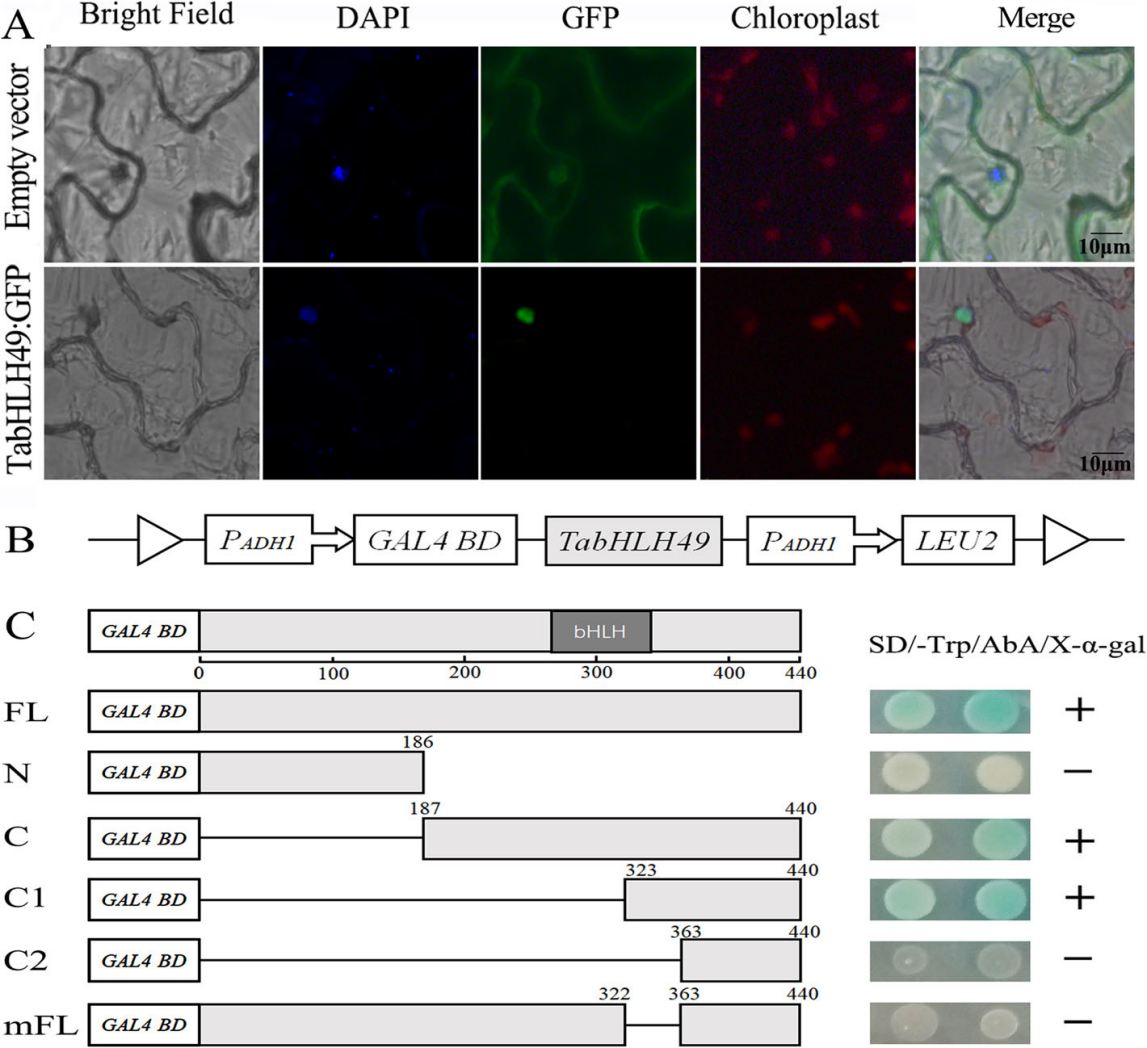
Subcellular localization (**a**) and transactivation assay (**b** and **c**) of TabHLH49 protein. **a** Subcellular localization of TabHLH49 in tobacco epidermal cells. GFP and TabHLH49:GFP under the control of the CaMV35S promoter were separately transiently expressed in tobacco epidermal cells. Scale bar = 10 μm. **b** Diagram of the pGBKT7 construct that expressed different truncated TabHLH49 proteins in yeast. **c** Transactivation activities of the intact or truncated TabHLH49. Fusion proteins of the GAL4 DNA-binding domain and different portions of TabHLH49 were transformed into Y2H Gold cells and grown on SD/−Trp/X-α-gal/AbA plates to assess their transactivation activities

According to the ProtComp prediction algorithm, the nuclear localization signal ‘VSCPKKRKRPSQ’ was present in the amino acid sequence of TabHLH49 (Fig. 1a). Therefore, TabHLH49 may be a nuclear localization protein. To test this reasoning, the subcellular localization of TabHLH49 was observed by coexpression of TabHLH49-GFP under the control of the 35S promoter. The recombinant vector was transformed into *Agrobacterium tumefaciens* strain GV3101 and then infiltrated into tobacco leaves. GV3101 cells transformed with the 35S:GFP vector were infiltrated into tobacco leaves as control. The results indicated that the green fluorescence of TabHLH49: GFP fusion protein was only in the nucleus of the tobacco epidermal cells, while the empty control was evenly distributed throughout the tobacco cells (Fig. 2a). This finding indicated that TabHLH49 is specifically localized to the nucleus.

The transactivation activity of the TabHLH49 protein was determined in Y2H Gold yeast cells. The expression of the reporter gene could not be activated when the C-terminus (amino acids 323–362) was absent, indicating that the amino acid sequences at the C-terminus (amino acids 323–362) are necessary for the transactivation activity of TabHLH49 (Fig. 2c).

### Expression patterns analysis of *TabHLH49* and *WZY2* in wheat

**Fig. 3:**
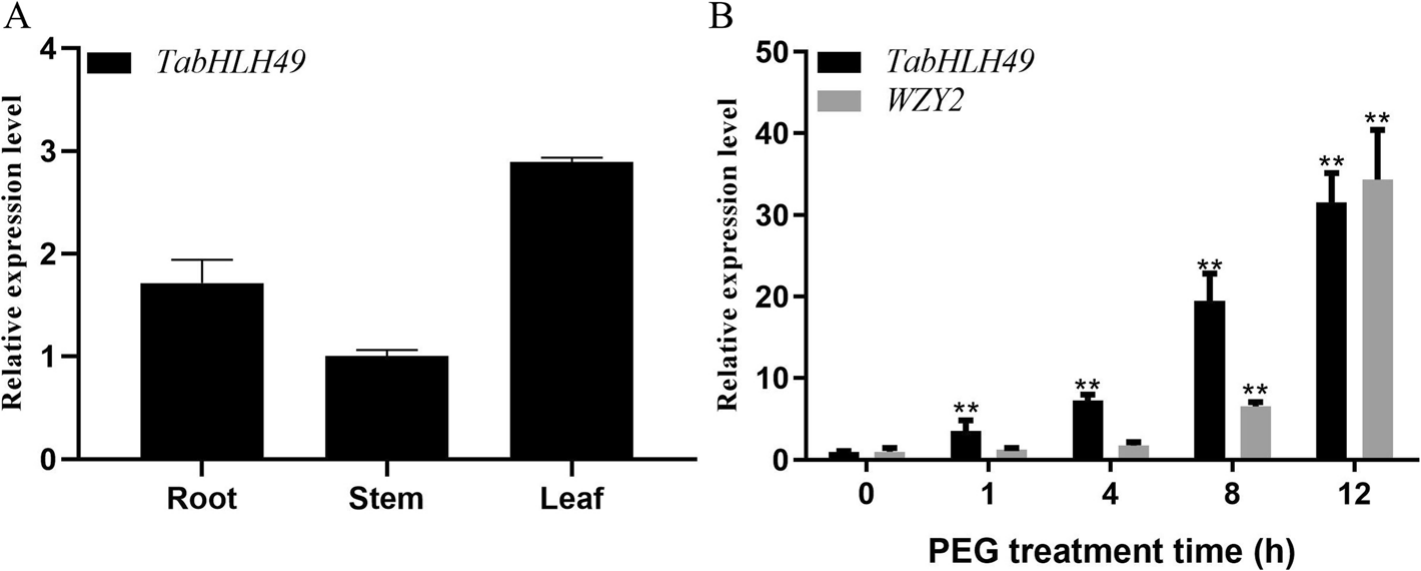
Expression patterns of *TabHLH49* and *WZY2* genes determined by real-time PCR. **a** Real-time PCR was used to analyze the expression levels of *TabHLH49* in different tissues. **b** Expression of *TabHLH49* and *WZY2* genes in response to 20% PEG 6000 treatment. The values are the mean ± SD from three samples, and significant differences are indicated as *p* < 0.05 (*) and *p* < 0.01 (**)

Real-time PCR was used to detect the expression of *TabHLH49* in different tissues of wheat (Fig. 3a). The results showed that *TabHLH49* was expressed in the roots, stems and leaves of 2-week-old seedlings. The expression of *TabHLH49* was abundantly accumulated in roots (1.71-fold) and leaves (2.89-fold) compared to stems.

Our previous studies showed that the expression of *WZY2* was highest in leaves during the wheat seedling stage [11]. Therefore, we selected the leaves of wheat seedlings to study the expression levels of *TabHLH49* and *WZY2* at different time points (0, 1, 4, 8 and 12 h) after PEG treatment (Fig. 3b). Real-time PCR results showed that the relative expression level of *TabHLH49* obviously increased over 1–12 h and reached the highest point, approximately a 31.6-fold change, at 12 h compared to 0 h. Interestingly, the expression of *WZY2* showed a similar tendency as *TabHLH49* but lagged behind *TabHLH49*. These results indicated that TabHLH49 may positively regulate the expression of *WZY2*.

### TabHLH49 is an upstream regulator of *WZY2*

**Fig. 4:**
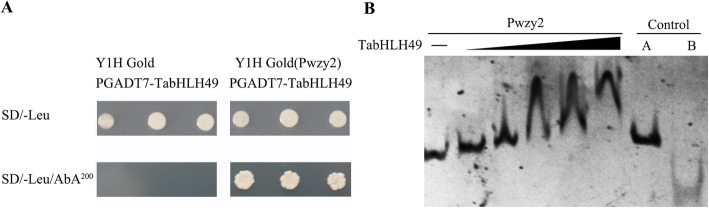
TabHLH49 interacts with the promoter of *WZY2* (Pwzy2). **a** Interaction of TabHLH49 with the promoter of *WZY2* in the Y1H assay. **b** EMSA was performed to analyze the interactions between His6-TabHLH49 and the *WZY2* promoter. Increasing amounts of TabHLH49 (0.1, 0.2, 0.4, 0.6 and 0.8 μM) were used. As negative controls, bovine serum albumin (BSA) instead of His6-TabHLH49 (control A) and (GAGCGTAACTGCCCACCACTCACTGGCTCACGCGCTGCCC) repeated tandem DNA fragment instead of the *WZY2* promoter (control B) were included in the binding assays

To examine the interaction between TabHLH49 and the *WZY2* promoter (Pwzy2), we performed yeast one-hybrid and EMSA assays. The yeast one-hybrid results showed that Y1H Gold yeast cells containing pGADT7-TabHLH49 could not grow on SD/−Leu/AbA^200^ selective medium. However, Y1H Gold (Pwzy2) bait yeast strains containing pGADT7-TabHLH49 were able to grow on SD/−Leu and SD/−Leu/AbA^200^ selective medium (Fig. 4a and Fig. S1). The EMSA experiments showed that the gel mobility of Pwzy2 changed after incubation with TabHLH49 protein (Fig. 4b and Fig. S2). These results indicated that TabHLH49 is an upstream regulator of *WZY2*.

### TabHLH49 positively regulates the expression of *WZY2* and improves the drought tolerance in wheat

**Fig. 5:**
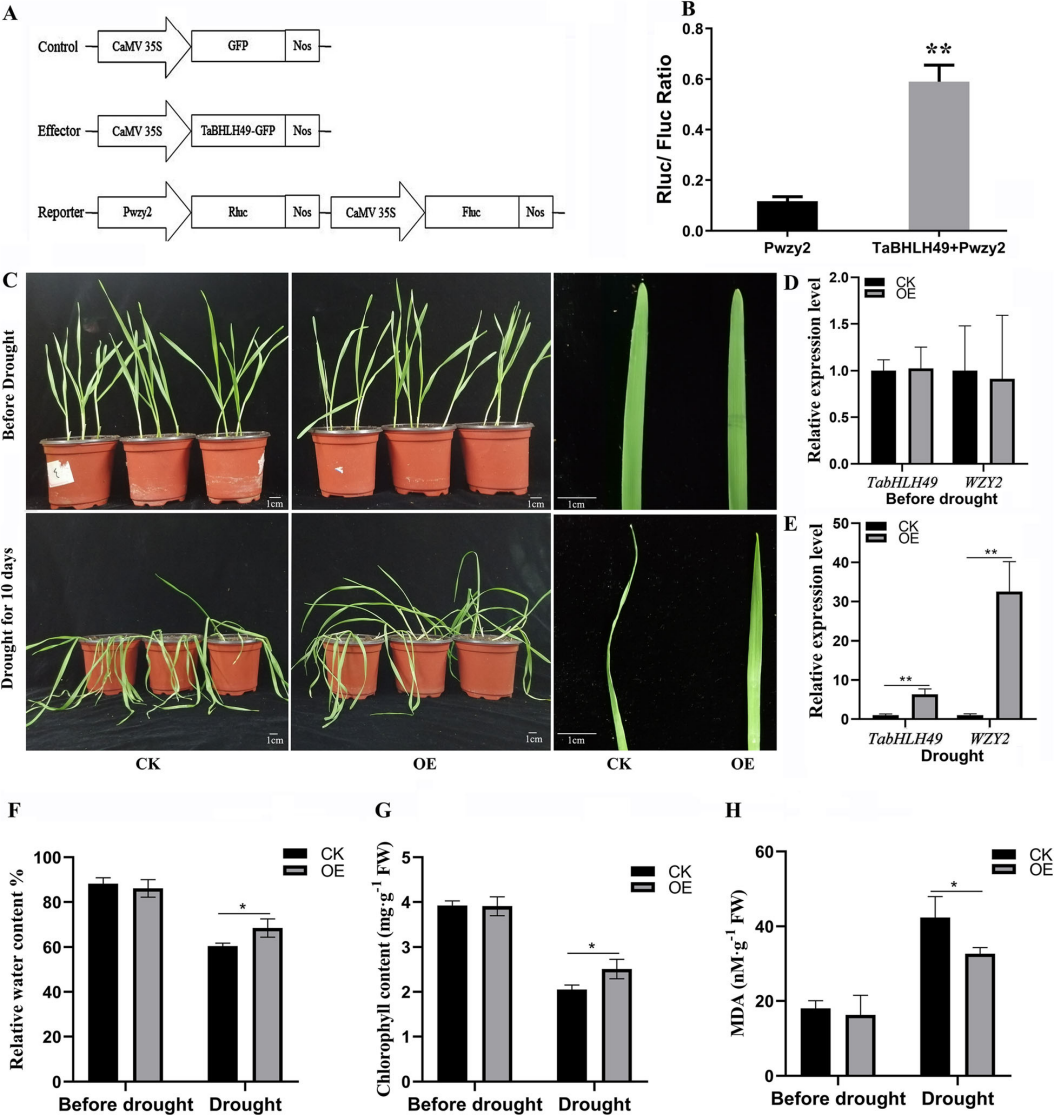
Transient expression assays of TabHLH49. **a** Schematic representations of pCAMBIA1302 empty vector (control), pCAMBIA1302-TabHLH49:GFP (effector) and reporter constructs used in the transient expression system. The Nos box indicates the terminator. **b** Transactivation effect of TabHLH49 on the promoter of *WZY2* (Pwzy2) shown by the dual-luciferase assay in tobacco leaves. The constructs described in (A) were transformed into *Agrobacterium tumefaciens* strain GV3101 and then infiltrated into tobacco leaves. The values of Rluc/Fluc are the mean ± SD from six samples, and significant differences are indicated as *p* < 0.05 (*) and *p* < 0.01 (**). (C-H) The phenotype (**c**), *TabHLH49* and *WZY2* gene expression levels (**d**-**e**), relative water content (**f**), chlorophyll content (**g**) and malondialdehyde content (**h**) of wheat leaves injected with *Agrobacterium* GV3101 containing pCAMBIA1302 empty vector (CK) and pCAMBIA1302:TabHLH49:GFP (OE) under normal growth conditions or drought stress conditions for 10 days. The values are the mean ± SD from three samples, and significant differences are indicated as *p* < 0.05 (*) and *p* < 0.01 (**)

To characterize the ability of *TabHLH49* to activate the *WZY2* gene, tobacco leaves were co-infiltrated with *A. tumefaciens* containing the 35S:TabHLH49:GFP vector together with a *Renilla luciferase* gene driven by the *WZY2* promoter. The Rluc/Fluc ratio was significantly increased by simultaneous TabHLH49:GFP overexpression compared to GFP expression (Fig. 5b).

To explore the function of TabHLH49 in wheat, *A. tumefaciens* GV3101 with 35S:TabHLH49:GFP (OE) and 35S:GFP (CK) were injected into wheat leaves for transient gene expression assays, as previously described [12, 13]. After natural drought for 10 d, the wheat leaves overexpressing GFP showed obvious wilting and curling; however, wheat that overexpressed TabHLH49:GFP showed better growth (Fig. 5c). Real-time PCR results showed that the expression of *TabHLH49* and *WZY2* was significantly increased in wheat leaves overexpressing TabHLH49:GFP compared to control after drought stress (Fig. 5d-e). We also determined the content of malondialdehyde (MDA), relative water content and chlorophyll content in the CK and OE lines (Fig. 5f-h). After drought stress, OE lines showed significantly lower MDA content, higher relative water content and higher chlorophyll content than CK lines.

**Fig. 6:**
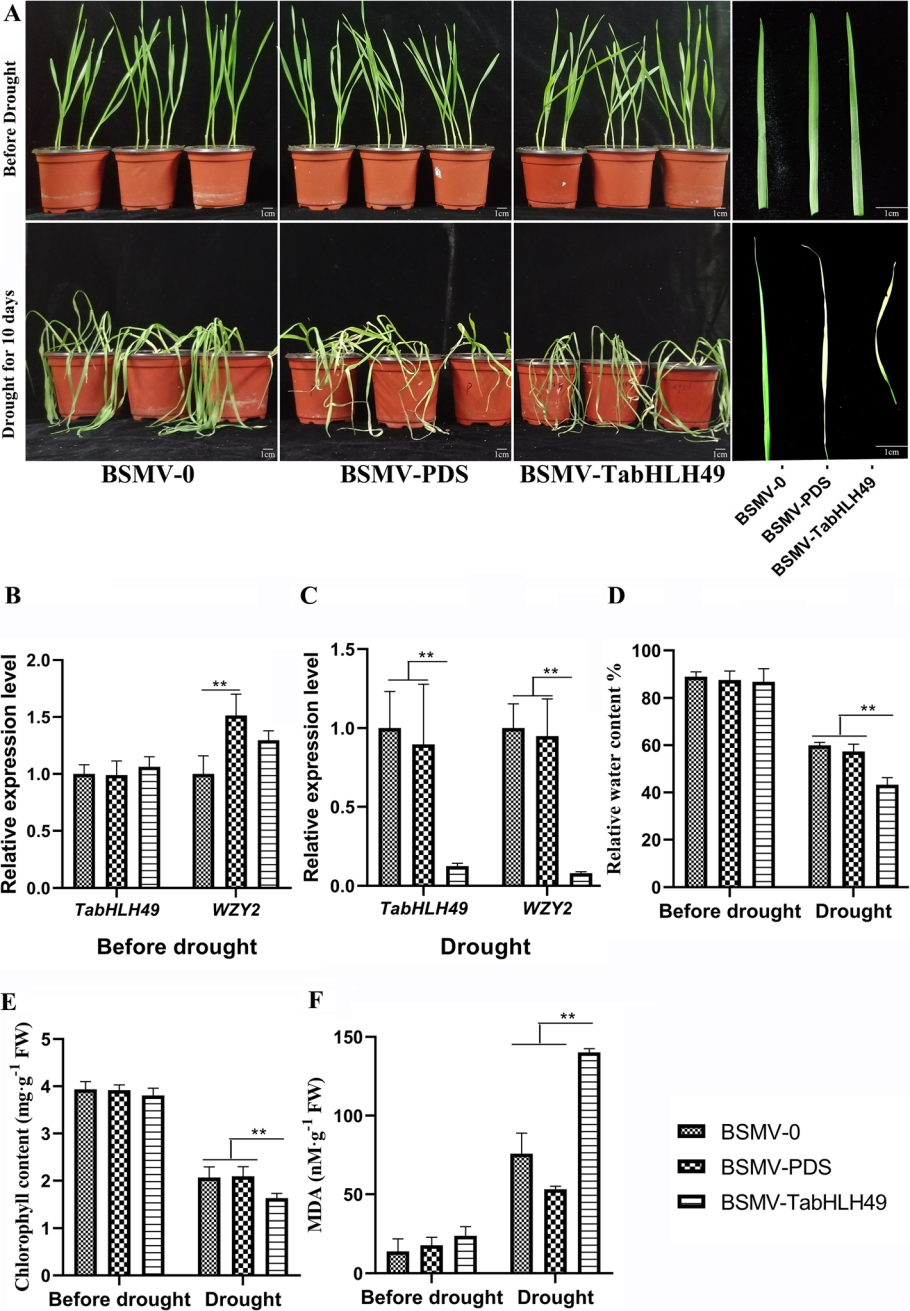
Virus-induced gene silencing (VIGS) of *TabHLH49* in wheat leaves. The phenotype (**a**), *TabHLH49* and *WZY2* gene expression levels (**b**-**c**), relative water content (**d**), chlorophyll content (**e**) and malondialdehyde content (**f**) of wheat leaves treated with BSMV-0 (empty vector), BSMV-PDS and BSMV-TabHLH49 under normal growth conditions or drought stress conditions for 10 days. The values are the mean ± SD from three samples, and significant differences are indicated as *p* < 0.05 (*) and *p* < 0.01 (**)

We also explored the function of the *TabHLH49* gene in wheat using the barley stripe mosaic virus-induced gene silencing (BSMV-VIGS) technique. The BSMV construct, BSMV-TabHLH49, carrying a 459 bp fragment from the *TabHLH49* cDNA sequence, was used to silence *TabHLH49* gene in wheat seed (Table S1). Wheat leaves photobleaching was observed in all plants infected with the BSMV-PDS construct at 10 days post inoculation (dpi), suggesting that the endogenous PDS gene was silenced (Fig. 6a). After natural drought for 10 d, the BSMV-TabHLH49 lines withered and curled more severely than the BSMV-0 and BSMV-PDS lines (Fig. 6a). Real-time PCR analysis showed that the transcript levels of *TabHLH49* and *WZY2* were reduced in the BSMV-TabHLH49 lines compared with the BSMV:0 and BSMV-PDS lines after drought stress (Fig. 6b-c). BSMV-TabHLH49 lines showed obviously higher MDA content, lower relative water content and lower chlorophyll content than BSMV-0 and BSMV-PDS lines after drought stress (Fig. 6d-f).

**Fig. 7:**
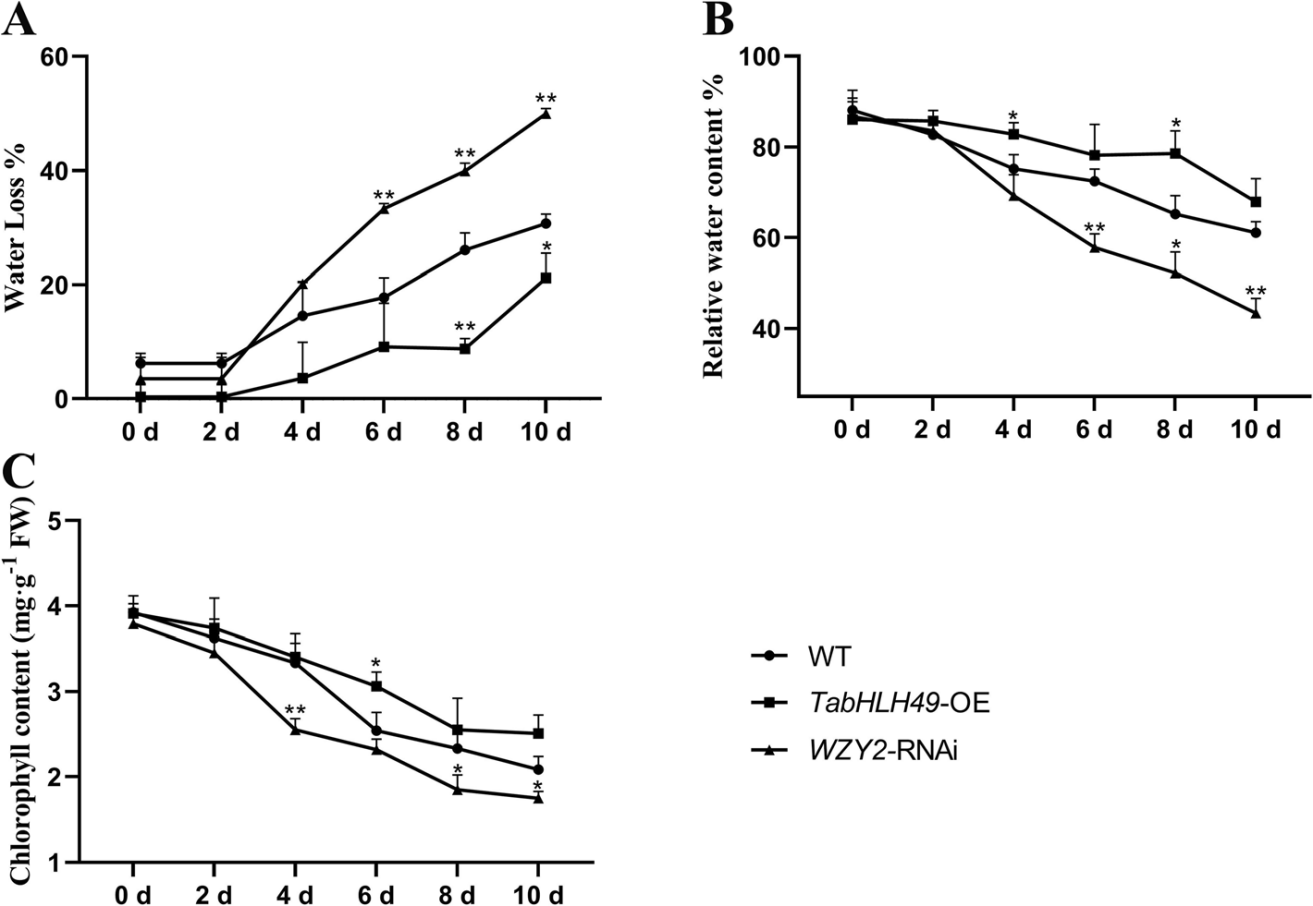
Determination of water loss rate, relative water content and chlorophyll content in *TabHLH49*-OE and *WZY2*-RNAi wheat lines. Water loss rate (**a**), relative water content (RWC%) (**b**) and chlorophyll content (**c**) of wheat leaves injected with *Agrobacterium* GV3101 containing pCAMBIA1302 empty vector (CK), pCAMBIA1302:TabHLH49:GFP (*TabHLH49*-OE) or *WZY2-*RNAi wheat lines. The values are the mean ± SD from three samples, and significant differences are indicated as *p* < 0.05 (*) and *p* < 0.01 (**)

In addition, we also measured the water loss rate, relative water content and chlorophyll content of wheat leaves from wild-type, *TabHLH49*-OE and *WZY2*-RNAi wheat lines. *TabHLH49*-OE wheat showed the highest relative water and chlorophyll contents and lowest water loss rate compared with the wild-type and *WZY2*-RNAi wheat lines under drought stress, while the *WZY2*-RNAi lines showed the lowest relative water and chlorophyll contents and the highest water loss rate (Fig. 7a-c). These results indicated that TabHLH49 positively regulated dehydrin *WZY2* gene expression to improve drought resistance in wheat.

## Discussion

### *TabHLH49* encodes stress-responsive bHLH transcription factor and positively regulates wheat drought tolerance

Transcription factors play an important function in regulating the drought response in plants. In *Arabidopsis*, many bHLH transcription factors are involved in the drought-induced stress response, such as *AtbHLH68* [14], *AtbHLH006* [15], *AtbHLH122* [16], *AtbHLH17* [17] and *AtbHLH92* [18]. However, only a few of bHLHs have been reported in wheat, such as *TabHLH1* [19] and *TabHLH39* [20], and the regulatory mechanism and biological functions of many other TabHLH proteins remain unclear. Here, we obtained a novel wheat bHLH transcription factor, TabHLH49, through the yeast one-hybrid (Y1H) assay with the dehydrin WZY2 promoter as bait. Phylogenetic analysis showed that TabHLH49 was highly homologous to AtbHLH049 and was a Myc-type bHLH transcription factor (Fig. 1b). *TabHLH49* was obviously induced by drought stress (Fig. 3b), and it enhanced the tolerance to drought stress (Fig. 5c). Compared with the BSMV-0 lines, the BSMV-TabHLH49 lines displayed reduced tolerance to drought stress (Fig. 6a). These results demonstrated that TabHLH49 encodes a novel bHLH transcription factor that positively regulates drought tolerance in wheat.

### TabHLH49 positively regulates dehydrin *WZY2* gene expression

Transcription factors regulate the expression of downstream genes by binding *cis*-acting elements of the promoter. In our previous study, we identified G-box elements in the promoter of *WZY2* that bind the bHLH transcription factor [21]. EMSA and Y1H assays illustrated that TabHLH49 protein can bind and interact with the promoter of *WZY2* (Fig. 4). The real-time PCR results indicated that the expression pattern of *WZY2* was similar to *TabHLH49* but lagged behind *TabHLH49* (Fig. 3b). Thus, TabHLH49 is a potential positive regulator of *WZY2* expression. A dual-luciferase assay also confirmed this result (Fig. 5b). In transient expression analysis, the *WZY2* expression level was significantly increased under drought stress in TabHLH49-OE wheat lines compared to CK (Fig. 5e). Similarly, the *WZY2* expression level was significantly reduced in BSMV-TabHLH49 lines (Fig. 6c). In addition, TabHLH49 protein showed no transcriptional activity in yeast when the C-terminus (amino acids 323–362) was deleted (Fig. 2c). These results indicate that TabHLH49 positively regulates dehydrin *WZY2* gene expression.

Taken together, the results presented in this study provide evidence that TabHLH49 positively regulates dehydrin *WZY2* gene expression to improve the drought resistance of wheat. We further need to develop stably overexpressed TabHLH49 to identify whether this transcription factor participates in stress, which will facilitate investigating the explicit role of TabHLH49 in the dehydrin signaling pathway.

## Conclusions

This study demonstrates that the transcription factor TabHLH49 positively regulates the expression level of the *WZY2* gene via binding to its promoter, thereby enhancing the drought resistance in wheat. Our research provides a better understanding of the regulatory mechanism of the *WZY2* genes in response to drought stress and offers potential strategies for further crop breeding.

## Methods

### Yeast one-hybrid screening of *T.aestivum* cDNA libraries

The 619 bp *WZY2* promoter (Pwzy2) was cloned into the pAbAi vector, and the recombinant plasmids were transformed into Y1H Gold yeast cells as bait [10]. Two-week-old wheat seedlings exposed to either 12 h high PEG 6000 (20%) or cold (4 °C) treatments were used to construct the cDNA library. The extraction of mRNA was performed with the MiniBEST Plant RNA Extraction kit (Taraka). First-strand cDNA was synthesized by the SMART cDNA Library Construction Kit (Taraka). All fragments were linked to the vector pGADT7-AD and transformed into yeast bait strain Y1H Gold (Pwzy2) following the Yeast Protocols Handbook (Clontech). The transformed stains were cultured on SD/−Leu/AbA^200^ medium. Yeast plasmid was extracted and amplified with the T7 and 3’AD sequencing primers. The amplicons were transformed into *Escherichia coli* and sequenced.

### Bioinformatics analysis of TabHLH49

The ORF of TabHLH49 was identified in the wheat genomic database of the Ensembl servers (http://plants.ensembl.org/Triticum_aestivum/Info/Index). The physiochemical characteristics were predicted by the ProtParam tool [22]. The basic helix-loop-helix conserved domain and the subcellular localization were found by the NCBI Conserved Domain database (https://www.ncbi.nlm.nih.gov/cdd) and ProtComp (http://linux1.softberry.com/), respectively. The Swiss-Model tool (https://swissmodel.expasy.org/) was used to predict the three-dimensional structure of the TabHLH49 protein. The phylogenetic tree was constructed by the neighbor-joining method with 1000 bootstrap replicates in MEGA 7.0 software [23]. The amino acid sequences of the wbHLH transcription factors were obtained from a previous report [24].

### Subcellular localization of TabHLH49

To study the subcellular localization of TabHLH49 protein, the *Agrobacterium* GV3101 carrying the recombinant plasmid pCAMBIA1302:TabHLH49:GFP and pCAMBIA1302 empty vector (control) were injected into *Nicotiana benthamiana* leaves. The fluorescence signal was observed in tobacco epidermal cells by fluorescence microscopy after culturing in the dark for 36 h at 28 °C.

### Analysis of TabHLH49 transactivation

A transcript activation activity assay was carried out as previously reported [8]. The full-length and truncated fragments of TabHLH49 were fused with GAL4 DNA-binding domain of the pGBKT7 plasmid and transformed into yeast Y2H Gold cells, and then grown on SD-Trp/X-α-gal/AbA plates to examine their growth and α-galactosidase activity.

### Plant materials and growth conditions

Wheat seeds of “*ZhengYin 1#*” were used throughout this study. Growth condition of wheat plants and 20% PEG6000 treatment were the same as that described in our previous study [25].

### RNA isolation and real-time PCR analysis

The protocol of RNA isolation and real-time PCR were described as our previous study [25]. All real-time PCR reactions were performed in triplicate to ensure reproducibility of the results.

### Expression of fusion TabHLH49 protein and purification

TabHLH49 was cloned into pET-28a vector, and recombinant plasmid was transformed into competent BL21 (DE3) cells. The expression of recombinant protein was induced with 1 mM isopropyl β-D-1-thiogalactopyranoside (IPTG) for 6 h after the recombinant *E. coli* density reached an OD_600_ of 0.6 in LB liquid medium containing with 50 μg/mL kanamycin at 37 °C. The cells were harvested by centrifugation and were resuspended in phosphate-buffered saline (PBS), followed by ultrasonic cell disruption for 30 min. The TabHLH49 protein was purified using ProteinIso™ Ni-NTA resin (TransGen) and detected by SDS-PAGE.

### Electrophoretic mobility shift assay

EMSA was performed with the LightShift® Chemiluminescent EMSA Kit (Thermo Fisher Scientific) following the manufacturer’s protocol. The 619 bp region of the *WZY2* promoter sequences were amplified using gene-specific primers (Table S1) and then purified and quantified. The *WZY2* promoter was reacted with the purified TabHLH49 protein at room temperature for 30 min. The complexes were then separated by native 6% polyacrylamide gel electrophoresis.

### Dual-luciferase assay

To investigate the transactivation role of TabHLH49 on the target *WZY2* promoter, a dual-luciferase assay was conducted according to previously reported [26]. The promoter of *WZY2* was cloned into the dual-luciferase report vector pC0390-RUC and transformed into *Agrobacterium* GV3101 and then injected together with either pCAMBIA1302:TabHLH49:GFP (effector) or pCAMBIA1302 empty vector (control) into tobacco leaves. After incubation for 3 d, the leaves were ground in liquid nitrogen and then mixed with passive lysis buffer at room temperature for 30 min. After centrifugation, the supernatant was collected and assessed for luciferase activity (RLuc/Fluc) using the Dual-Luciferase® Reporter Assay System (Promega).

### Transient expression analysis of TabHLH49

The *Agrobacteria* GV3101 transformed with the pCAMBIA1302:TabHLH49:GFP and pCAMBIA1302 empty vector (control) were injected into wheat leaves for transient gene expression assays, as previously described [12, 13]. Fresh *Agrobacteria* were grown overnight in yeast extract broth (YEB) medium with rifampicin, carbenicillin, and spectinomycin. After centrifugation, the bacterial pellets were resuspended in an infiltration buffer containing 10 mM MES, 10 mM MgCl_2_, and 400 μM acetosyringone at an optical density of OD_600_ = 2.0. For wheat infiltration, the fully expanded secondary leaf of wheat seedlings was infiltrated using a 1 mL syringe without a needle. Plant phenotypes were examined between 5 and 14 d after infiltration.

### BSMV-mediated *TabHLH49* gene silencing

In vitro transcription of viral RNA and plant inoculations were performed as previously described [27]. Four plants of wheat were infected with BSMV-PDS (positive control) or BSMV-TabHLH49 construct, with inoculation with BSMV:0 (negative control) was used as a control in each experiment. This experiment was repeated three times, and the data were averaged.

### Determination of malondialdehyde (MDA), water loss, relative water content and chlorophyll content

Determination of the malondialdehyde (MDA) content, the water loss rate, the relative water content and the chlorophyll content of wheat leaves after drought stress was performed as previously described [11, 28, 29].

## Supplementary information

**Additional file 1: Table S1.** Specific primers used in the study.

**Additional file 2: Figure S1.** Raw images of Fig. 4a.

**Additional file 3: Figure S2.** Raw images of Fig. 4b.

## Data Availability

The datasets generated and analysed during the current study are available in the UniProt database with the link of https://www.uniprot.org/, under the accession number B0LXL4 and A0A3B6RCI9, respectively. The sequences of *WZY2* promoter can be found in the additional files of our previous study with the link of 10.1080/15592324.2019.1678370.

## Abbreviations

bHLH: Basic helix-loop-helix transcription factor
Pwzy2: Promoter of the dehydrin *WZY2* gene;
ABA: Abscisic acid
EMSA: Electrophoretic mobility shift assay
Rluc: Renilla luciferase
Fluc: Firefly luciferase
AbA: Aureobasidin A
Y1H: Yeast one-hybrid
IPTG: Isopropyl β-D-1-thiogalactopyranoside
MDA: Malondialdehyde
VIGS: Virus-induced gene silencing
