# Supplementary material for: Transcription factor TabHLH49 positively regulates dehydrin WZY2 gene expression and enhances drought stress tolerance in wheat
Source: BMC Plant Biol. 2020 Jun 5;20:259. doi: 10.1186/s12870-020-02474-5 (PMC7275420; doi:10.1186/s12870-020-02474-5)
Supplement: Supplementary file 1 — Additional file 1: Table S1. Specific primers used in the study. [file 12870_2020_2474_MOESM1_ESM.docx]

**Transcription factor TabHLH49** **positively regulates dehydrin *WZY2* gene expression and enhances drought stress tolerance in wheat**

Hao Liu^1,†^, Ying Yang^1,2,†^, Dandan Liu^3,†^, Xiaoyu Wang^1,4^, Linsheng Zhang^1,^*

1 College of Life Science/State Key Laboratory of Crop Stress Biology for Arid Areas, Northwest A&F University, Yangling 712100, China.

2 College of Nursing, Weinan Vocational&Technical College, Weinan 714000, China.

3 School of Agriculture, Yunnan University, Kunming 650000, China.

4 Institute of Evolution & Marine Biodiversity, Ocean University of China, Qingdao 266000, China

*Correspondence: [linszhang@nwsuaf.edu.cn](mailto:linszhang@nwsuaf.edu.cn).

†These authors contributed equally to this work.

**Table S1. Specific primers used in the study.**

| Primer name | Sequence (5'-3') |
| --- | --- |
| pABAi-Pwzy2 F | AGATCCCCGGGTACCGAGCTCGTCGTGGCTATGGTGATGC |
| pABAi-Pwzy2 R | AAATGATGAATTGAAAAGCTTCTTGCACAGGTGCTGATCT |
| Clone-TabHLH49 F | CGCAGATTTCGGTCTTCCAG |
| Clone-TabHLH49 R | TCGGTTGCGGAATCTATTGC |
| qCR-WZY2 F | GAGTACCAGGGACATCAGCA |
| qCR-WZY2 R | ATGCCATCATCCTCAGACGA |
| qCR-TabHLH49 F | CTGCATCGTCTACCTTTGGC |
| qCR-TabHLH49 R | CTCATCCAACGAAACGGAAT |
| qCR-Actin F | ACCCAACCAGAAACAGCAAC |
| qCR-Actin R | TGTAGGTGGCAAAGGTCTC |
| PGADT7-TabHLH49 F | GCCATGGAGGCCAGTGAATTCATGGAGACGAATGAGAAGA |
| PGADT7-TabHLH49 R | CAGCTCGAGCTCGATGGATCCTCAAGGTTTATCATT |
| EMSA-Pwzy2 F | GTCGTGGCTATGGTGATGC |
| EMSA-Pwzy2 R | CTTGCACAGGTGCTGATCT |
| pET28a-TabHLH49 F | ATGGGTCGCGGATCCGAATTCATGGAGACGAATGAGAAG |
| pET28a-TabHLH49 R | GTGGTGGTGGTGGTGCTCGAGTCAAGGTTTATCATTTGA |
| 1302-TabHLH49 F | CGGGGGACTCTTGACCATGGAGACGAATGAGAAGA |
| 1302-TabHLH49 R | TACTAGTCAGATCTACCATGGAGGTTTATCATTTGA |
| LUC-Pwzy2 F | CTGCAGGTCGACGGATCCCCGGGGTCGTGGCTATGGTGATGC |
| LUC-Pwzy2 R | GGTGGACTCCTCTTAGAATTCCTTGCACAGGTGCTGATCT |
| BSMV-TabHLH49 F | ATATTAATTAACATGTACAAGAAGGGAGCCA |
| BSMV-TabHLH49 R | TAGCGGCCGCGCCAAAGGTAGACGATGCAG |
