# Supplementary figures and images for: Transcription factor TabHLH49 positively regulates dehydrin WZY2 gene expression and enhances drought stress tolerance in wheat
Source: BMC Plant Biol. 2020 Jun 5;20:259. doi: 10.1186/s12870-020-02474-5 (PMC7275420; doi:10.1186/s12870-020-02474-5)

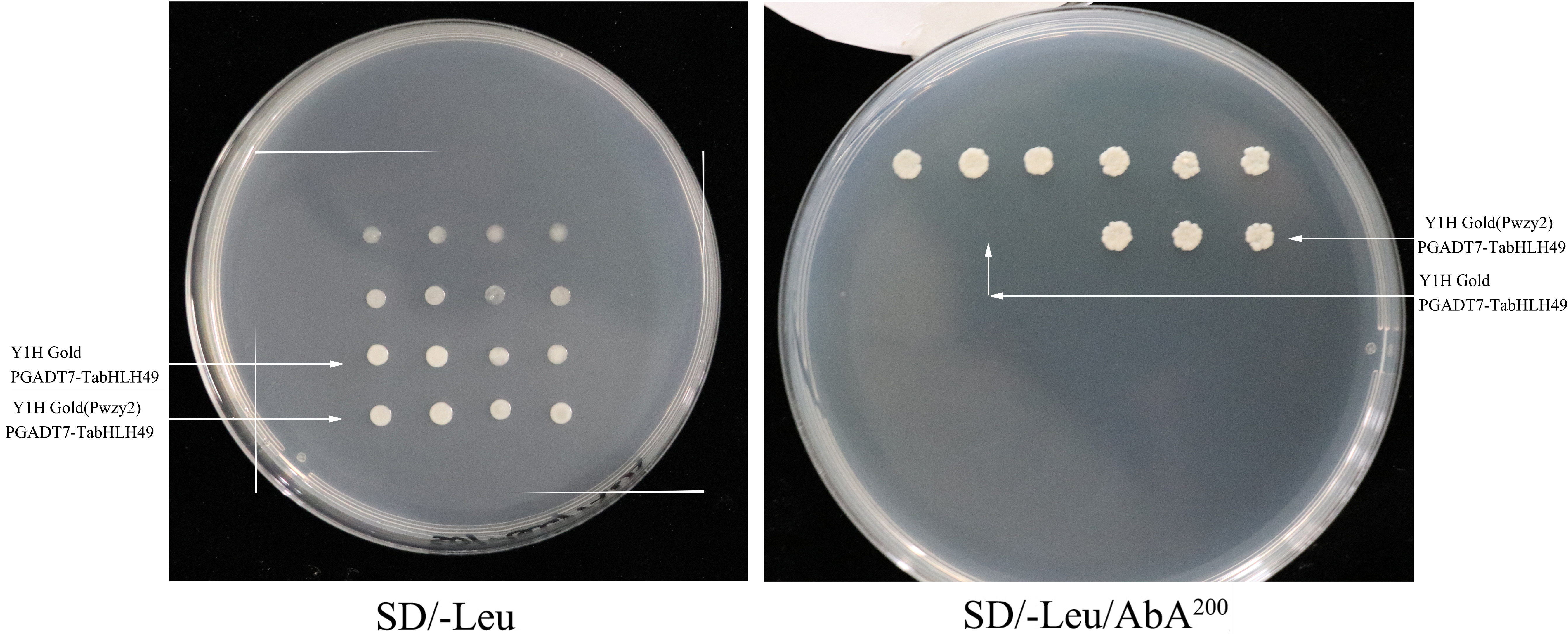

Supplement: Supplementary file 2 — Additional file 2: Figure S1. Raw images of Fig. 4a. [file 12870_2020_2474_MOESM2_ESM.tif]

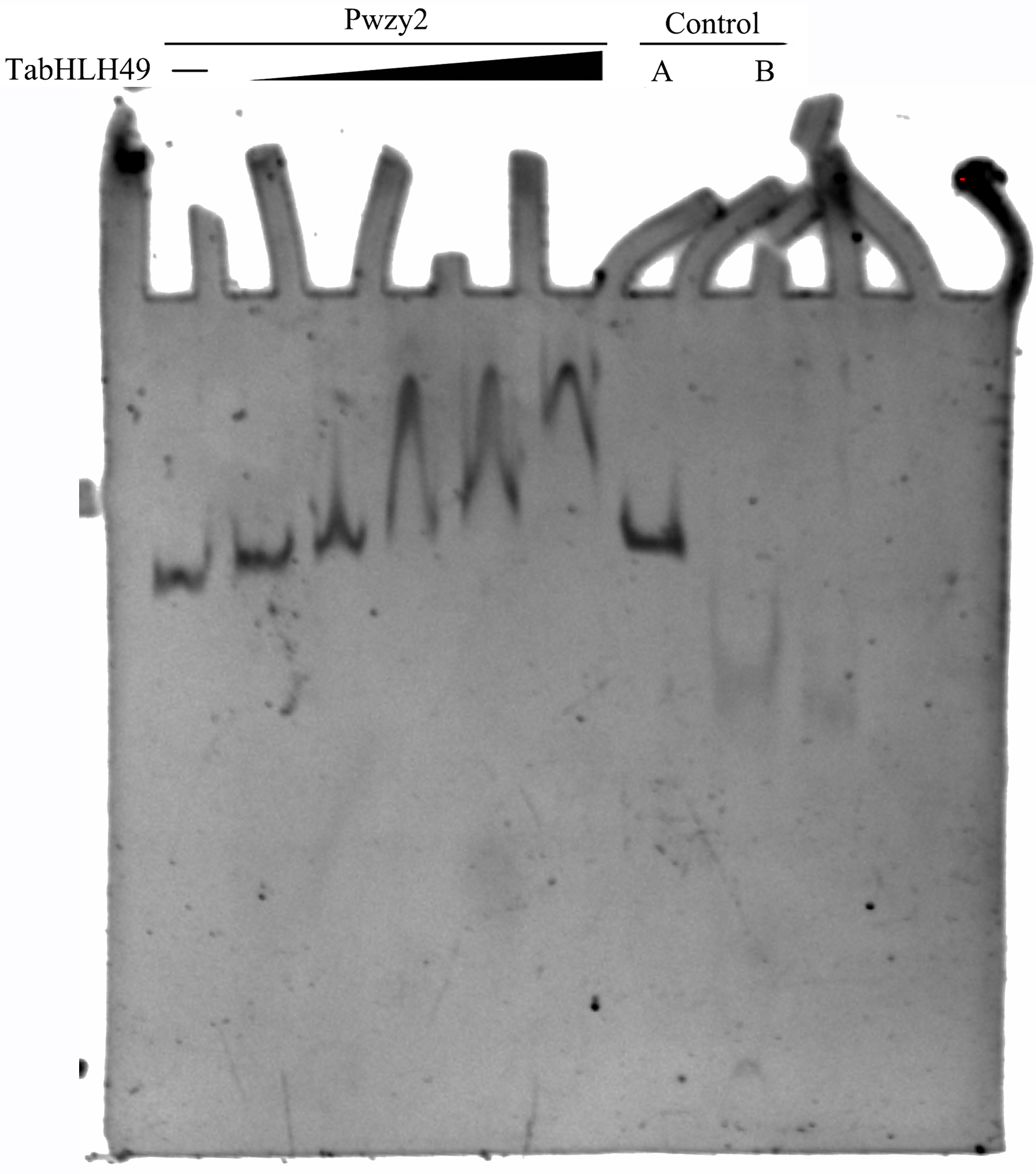

Supplement: Supplementary file 3 — Additional file 3: Figure S2. Raw images of Fig. 4b. [file 12870_2020_2474_MOESM3_ESM.tif]
